# Detection of acute myeloid leukemia and remission states using heterogeneous flow cytometry data

**DOI:** 10.3389/fonc.2025.1638074

**Published:** 2025-09-30

**Authors:** Renjun Bao, Ming Feng, Mian Wang, Yunkai Liu, Liang Hu, Yonghua Yao

**Affiliations:** ^1^ Department of Hematology, Shidong Hospital of Shanghai Yangpu District, Shanghai, China; ^2^ School of Computer Science and Technology, Tongji University, Shanghai, China; ^3^ Laboratory Diagnosis Department, Shanghai Kingmed Center for Clinical Laboratory Co., Ltd., Shanghai, China

**Keywords:** acute myeloid leukemia, flow cytometry, machine learning, feature importance analysis, diagnostic model

## Abstract

**Introduction:**

Acute myeloid leukemia (AML) is a hematological malignancy that requires accurate diagnosis and continuous monitoring to guide effective treatment. Flow cytometry is widely used because it enables the detection of minimal residual disease. However, current methods often rely on uniform marker panels, overlooking the heterogeneity that arises when different markers or staining protocols are used across patients. In addition, remission states are frequently neglected, despite their clinical importance for disease management and prognosis.

**Methods:**

To address these challenges, we developed a machine learning–based classification framework that integrates heterogeneous flow cytometry data. A dataset comprising 53 markers was collected, and six different machine learning classifiers were trained to distinguish between AML, complete remission (AML-CR), and normal samples. Model performance was evaluated using accuracy, precision, recall, F1 score, and area under the ROC curve (AUC).

**Results:**

Among the classifiers evaluated, the Random Forest model demonstrated the highest performance, achieving an accuracy of 94.92%, an F1-score of 94.13%, a precision of 94.58%, a recall of 93.74%, and an AUC of 94.83%. These results indicate that machine learning can effectively classify AML and remission states from heterogeneous flow cytometry data.

**Discussion:**

This study highlights the value of machine learning in overcoming limitations of traditional flow cytometry analysis. By accommodating marker heterogeneity and incorporating remission states, the proposed framework provides a more robust and clinically relevant tool for AML diagnosis and monitoring. The findings suggest that machine learning models, particularly Random Forest, hold strong potential for improving precision in hematological diagnostics. The code for this study is publicly available at https://zenodo.org/records/15110287.

## 1 Introduction

Acute myeloid leukemia (AML) is a malignant clonal disease originating from the abnormal proliferation and differentiation of hematopoietic stem cells. It represents the most prevalent form of adult leukemia (1). Significant improvements in AML prognosis have been achieved through advancements in chemotherapy, targeted therapy, transplantation techniques, CAR-T therapy, and the ongoing refinement of supportive care (2–4). Flow cytometry, which utilizes specific antibodies to label surface antigens on leukemia cells, is capable of identifying and quantifying as few as 0.01% leukemia cells. It has emerged as a critical tool for the diagnosis and monitoring of AML, widely applied in the analysis of diverse cell populations (5, 6) and the assessment of minimal residual disease (MRD) to evaluate disease prognosis (7, 8). However, flow cytometry relies heavily on manual operation, which is associated with significant drawbacks, including time-consuming procedures, high subjectivity, and inconsistent results, potentially leading to missed diagnoses or misdiagnoses (9). Therefore, investigating the potential for automated diagnosis based solely on flow cytometry data and developing an intelligent diagnostic system that is automated, precise, and broadly applicable holds significant clinical value for early diagnosis and treatment.

In recent years, machine learning (ML) has achieved remarkable advancements in intelligent medical diagnosis, particularly in disease classification, prediction, and personalized treatment (10–12). ML excels at automatically learning from large-scale datasets and uncovering underlying patterns (13), offering unparalleled advantages over traditional methods, especially when processing complex and high-dimensional biomedical data such as flow cytometry data (14). However, existing models may not be directly applicable to real-world flow cytometry diagnostic scenarios due to the unstandardized nature of flow cytometry data, which often fails to meet the input requirements of these models. Specifically, in practical settings, the performance limitations of flow cytometry instruments necessitate the use of multiple panels to obtain comprehensive data for a single patient. For instance, the same marker may be labeled with different fluorescent dyes, leading to variations in the measured values. Such discrepancies are rarely encountered in publicly available standardized datasets, where each sample typically employs a consistent combination of markers and dyes (15). Furthermore, existing studies on flow cytometry datasets frequently overlook the analysis of patients with complete acute myeloid leukemia remission (AML-CR), which oversight limits the comprehensive understanding of patient data distribution, impairs the evaluation of treatment efficacy, and hinders the monitoring of disease relapse risk.

To address this issue, we collected a dataset from real-world diagnostic scenarios, encompassing samples with diverse combinations of antibodies and dyes. A key advantage of this dataset is its inclusion of AML-CR samples, enabling us to investigate variations in cellular populations across different disease stages. Subsequently, we calculate the statistical properties of each marker to standardize the samples into a consistent format. This step preserves the distribution information of markers while ensuring compatibility with the input requirements of ML models. Finally, ML algorithms are employed to automatically extract feature information from the standardized flow cytometry data and construct robust classification models.

Specifically, this study collected flow cytometry data from 59 AML patients, 34 AML-CR patients, and 101 bone marrow flow cytometry-normal (Norm) patients, encompassing the expression profiles of various cell surface markers. Utilizing multiple ML algorithms for feature extraction, selection, and modeling, we developed a diagnostic model capable of distinguishing among the three patient groups. The model underwent rigorous feasibility analysis, performance validation, and comprehensive evaluation. Extensive experimental results demonstrate the efficacy of the proposed method in AML diagnosis. Furthermore, we conducted additional analysis to assess the importance of markers within the model. On one hand, the model’s findings align with clinical knowledge, mutually reinforcing each other. On the other hand, the identification of potentially significant features may offer novel insights into disease mechanisms. This approach not only enhances our understanding of the immunological characteristics of AML but also equips clinicians with more scientific and efficient diagnostic tools.

## 2 Methods

### 2.1 Study population

This retrospective study analyzed flow cytometry data from patients treated at Shidong Hospital, Yangpu District, Shanghai, between January 2019 and October 2024. The study included samples from patients with acute myeloid leukemia (non-M3 type, AML), AML in complete remission (AML-CR), and those with normal bone marrow flow cytometry results (Norm). The normal group comprised patients with cytopenia or cytosis caused by non-neoplastic conditions, including nutritional anemia, immune thrombocytopenia, and primary thrombocythemia. Inclusion criteria: The study subjects are AML patients aged between 18 and 70 years. The diagnosis and classification of leukemia are based on the World Health Organization 5th Edition Classification of Hematologic and Lymphoid Tumors (16), with comprehensive evaluation considering clinical manifestations, morphology, cytogenetics, and molecular results (17). Complete remission of bone marrow after treatment is assessed according to the 4th Edition of Diagnostic and Efficacy Criteria for Hematologic Diseases, with a blast cell percentage of <5% defined as complete remission. Exclusion criteria: Patients with other hematologic disorders, severe infections, or other systemic diseases that may affect flow cytometry results, as well as cases with incomplete or obviously abnormal data.

**Figure 1:**
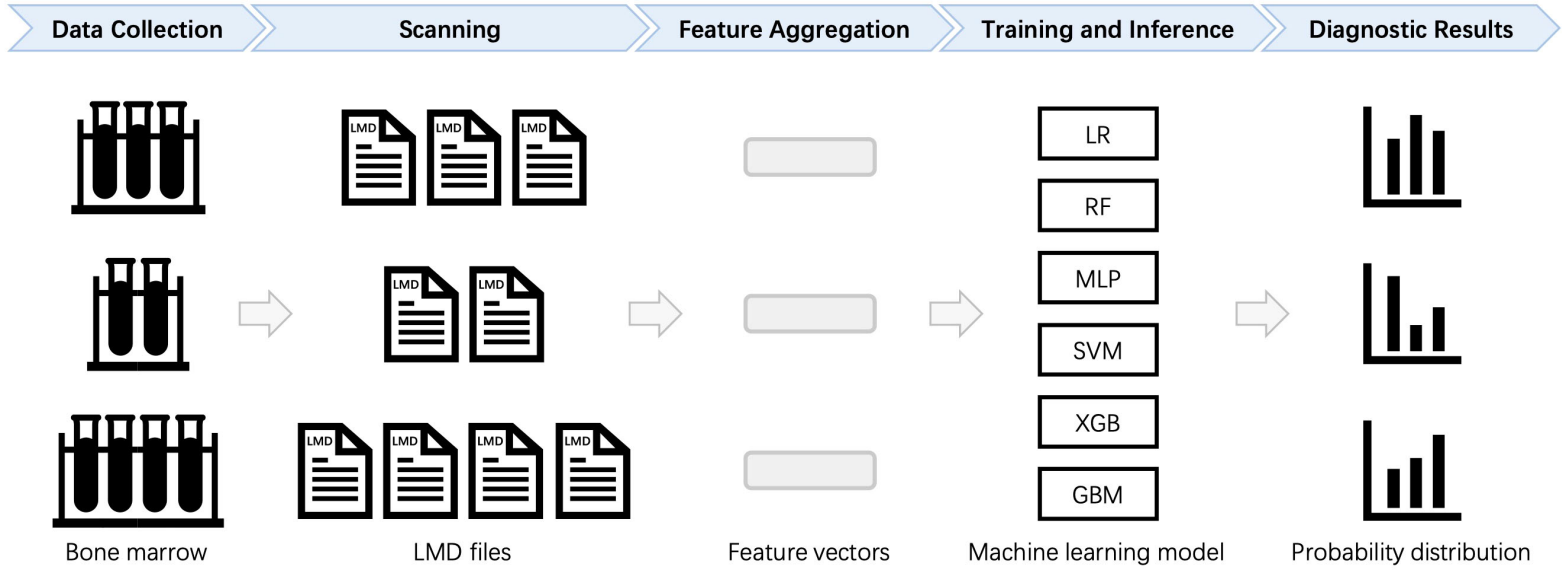
Automated diagnostic workflow for flow cytometry data analysis. For each patient, multiple LMD files are processed to calculate statistical features, which are then aggregated into a standardized format. The formatted data are subsequently input into a machine learning (ML) model for training and classification. LR, Logistic Regression; RF, Random Forest; MLP, Multilayer Perceptron; SVM, Support Vector Machine; XGB, Extreme Gradient Boosting; GBM, Gradient Boosting Machine.

Based on the above criteria, a total of 59 AML samples, 34 AML-CR samples, and 101 Norm samples are included in the study. The dataset comprised 53 distinct markers, as detailed in **Table 1**, which are utilized for subsequent feature engineering to extract and optimize classification features. The data are randomly split into training and testing sets at a ratio of 7:3 while maintaining the proportional distribution of each category in both sets. The complete workflow for sample screening and data processing is illustrated in **Figure 1**. The automatic diagnostic process of the flow cytometry-based model begins with data collection, where patient samples are acquired and raw flow cytometry data are generated. These data then undergo scanning, involving preprocessing and quality control to ensure accuracy and consistency. Next, during feature aggregation, relevant cellular features are extracted and combined to form comprehensive representations of each sample. The aggregated features are used in the training and inference phase, where the machine learning model is trained on labeled datasets and subsequently applied to new patient data for prediction. Finally, the model produces diagnostic results that assist clinicians in making decisions.

**Table 1:** List of markers used in flow cytometry analysis.

CD10-FITC	CD10-PE	CD117-PC5	CD11B-FITC	CD123-PE
CD13-PE	CD138-APC	CD138-PE	CD14-ECD	CD15-PC5
CD16-ECD	CD16-PE	CD19-ECD	CD19-PC5	CD2-PC5
CD20-ECD	CD23-PE	CD3-ECD	CD33-PE	CD34-ECD
CD34-PC5	CD36-FITC	CD38-FITC	CD4-APC	CD4-PE
CD41-ECD	CD45-KO	CD45-PC7	CD5-FITC	CD5-PC5.5
CD56-PC5	CD56-PC5.5	CD56-PE	CD57-FITC	CD64-PE
CD7-PC5	CD7-PE	CD71-FITC	CD79B-PC5.5	CD8-FITC
CD9-FITC	CKAPPA-FITC	CLAMBDA-PE	FMC7-FITC	FS-LIN
FS-PEAK-LIN	HLA-DR-ECD	HLA-DR-FITC	KAPPA-FITC	LAMBDA-PE
SS-LIN	TCRAB-FITC	TCRGD-PE		

Since the values measured for the same marker vary under different dyes, we treat each marker-dye pair as a distinct marker here.

### 2.2 Data selection

Fresh bone marrow samples (3 − 5mL), anticoagulated with heparin or EDTA, are collected and thoroughly mixed before storage at room temperature. The leukocyte count is determined using an automated hematology analyzer. Based on the leukocyte count, the sample is either diluted with PBS or concentrated by centrifugation at 1700 rpm to adjust the leukocyte concentration to 1 × 10^7^/mL. Tubes are prepared according to the sample and the specific antibody panel. For membrane staining, a pre-prepared antibody cocktail is added to each tube based on the selected antibody combination. The sample is thoroughly mixed (at least 5 inversions), and the calculated volume of the diluted or concentrated sample is added to the bottom of the tube. After gentle mixing, the sample is incubated in the dark for 15–20 minutes to ensure optimal staining efficiency. Subsequently, red blood cell lysis buffer is added, followed by an additional 10-minute incubation in the dark until complete lysis is achieved. Centrifuge the sample at 1700 rpm for 5 minutes, then discard the supernatant. The pellet is resuspended in 2 mL of PBS, mixed thoroughly, and centrifuged again at 1700 rpm for 5 minutes. After discarding the supernatant, 600 µL of 1% paraformaldehyde fixation solution is added to resuspend the cells, which are then subjected to flow cytometry analysis. For intracellular staining, the above steps are followed according to the reagent manufacturer’s instructions. For detection of surface or cytoplasmic immunoglobulin light chains, the sample is washed three times with PBS before antibody addition. Data acquisition is performed on a Navios 10 COLORS/3 LASER flow cytometer, ensuring at least 5 × 10^5^ events are collected per sample. The antibody panel included surface markers such as CD34, CD38, CD45, and CD117. All data are stored in LMD file format.

### 2.3 Data pre-processing and feature engineering

**Figure 2:**
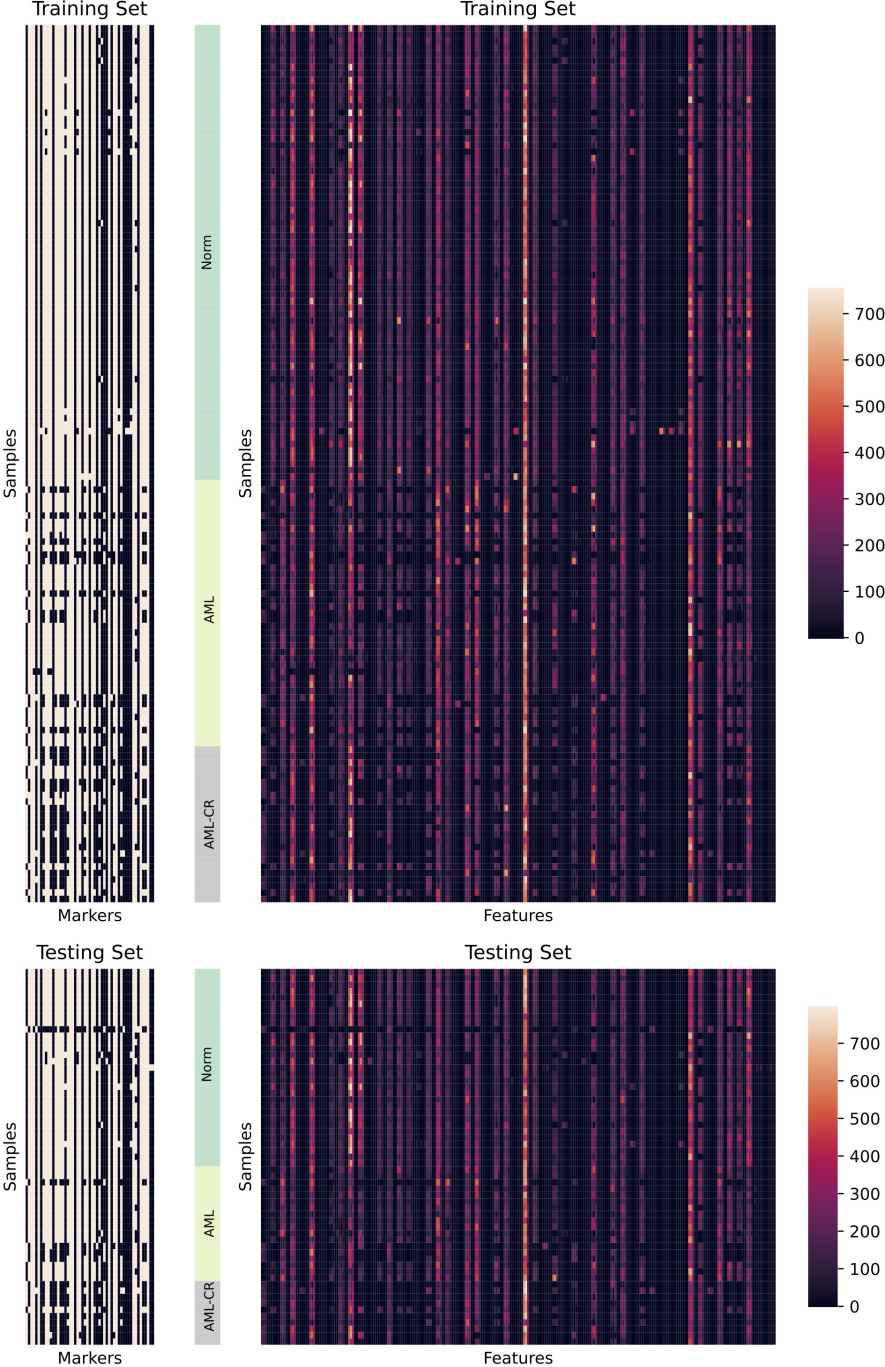
A detailed visualization of the dataset is presented. The left panel illustrates the distribution of markers across different samples, while white indicates the presence of corresponding markers and black indicates their absence. The right panel displays the distribution of features extracted from different samples. Here, Markers refers to the 53 cell surface markers analyzed, while Features represents the 265 statistical features derived from the 53 markers.

Each sample corresponds to tens of thousands of cells, and each cell carries multiple marker results. In clinical diagnosis, physicians often focus on the distribution patterns of these markers across different cell populations. Simply averaging the marker values for all cells may overlook important distribution characteristics. To address this, we incorporated additional statistical measures, including mean, standard deviation (std), median, skewness, and kurtosis, to capture the variability and asymmetry of marker distributions more comprehensively. These statistical measures effectively capture the distributional differences of markers across cell populations. The mean reflects the overall expression level of a marker, while the standard deviation quantifies variability among cells. The median reduces the influence of extreme values, skewness reveals distribution asymmetry, and kurtosis indicates the sharpness of the distribution or the presence of outliers. The formulas for calculating skewness and kurtosis are shown in Equations 1, 2, respectively.


(1) $$Skewness=\frac{1}{n}\sum_{i=1}^{n}\left[{\left(\frac{X_{i}-\mu }{\sigma }\right)}^{3}\right]$$

(2) $$Kurtosis=\frac{1}{n}\sum_{i=1}^{n}\left[{\left(\frac{X_{i}-\mu }{\sigma }\right)}^{4}\right]$$

where $X_{i}$ is the i th data point, μ is the sample mean, σ is the sample standard deviation, and n is the total number of samples. Therefore, the 53 marker values of all cells from each patient are transformed into a 265-dimensional feature vector, where each marker is represented by the five aforementioned distributional features. This transformation enables a more comprehensive representation of marker distribution across cells. The numerical distribution of the transformed data in the training and test sets is illustrated in **Figure 2**.

### 2.4 Model establishment and evaluation

The flow cytometry data is randomly split into training and testing sets in a 7:3 ratio. To ensure a comprehensive evaluation, this study employs six widely used ML algorithms, encompassing various classical approaches. These include linear models [Logistic Regression, LR (18)], ensemble methods [Random Forest, RF (19); Extreme Gradient Boosting, XGBoost (20); Gradient Boosting Machine, GBM (21)], neural networks [Multilayer Perceptron, MLP (22)], and support vector machines [Support Vector Machine, SVM (23)]. Each algorithm represents a distinct learning paradigm: linear models effectively capture linear relationships and are suitable when interpretability and simplicity are important, ensemble methods enhance predictive performance by aggregating multiple weak learners and work well for complex, nonlinear, and noisy data, neural networks excel at modeling complex nonlinear patterns and are particularly effective with large datasets and intricate feature interactions, support vector machines are well-suited for high-dimensional classification tasks, especially when the classes are separable with clear margins. All models are implemented using scikit-learn==1.6.1 in Python 3.10. In the training set, all flow cytometry data are divided into five parts, and five-fold cross-validation is applied. This strategy helps assess the stability and generalization ability of the model by cycling through each subset as the validation set. During cross-validation, grid search is used to optimize the hyperparameters. The specific parameter search space for each method is shown in **Table 2**. The evaluation metrics include accuracy, F1-score, precision, recall, and area under the curve (AUC). These metrics collectively reflect the model’s performance in the classification task, with particular significance for F1-score and AUC when handling imbalanced data. In addition, to analyze the contribution of features to the prediction, shap==0.46.0 (24) is used to estimate feature importance. Based on the concept of Shapley values, SHAP assigns an importance value to each feature, thereby helping to explain the model’s decision-making process and enhancing the model’s interpretability and reliability.

**Table 2:** Hyperparameter search space for machine learning (ML) models.

Model	Hyperparameters
Random Forest (RF)	n_estimators: [50, 100, 200] max_depth: [10, 20, 30] min_samples split: [2, 5, 10] min_samples leaf: [1, 2, 4] max_features: [‘sqrt’, ‘log2’, None]
Support Vector Machine (SVM)	C: [0.1, 1, 10] kernel: [‘linear’, ‘rbf’, ‘poly’] gamma: [‘scale’, ‘auto’] degree: [3, 5] class_weight: [None, ‘balanced’]
Multilayer Perceptron (MLP)	hidden_layer_sizes: [(50), (100), (50, 50)] activation: [‘relu’, ‘tanh’] solver: [‘adam’, ‘sgd’] alpha: [0.0001, 0.001, 0.01] learning_rate: [‘constant’, ‘invscaling’, ‘adaptive’]
Logistic Regression (LR)	C: [0.01, 0.1, 1, 10, 100] penalty: [‘l1’, ‘l2’] solver: [‘liblinear’, ‘saga’] max_iter: [100, 200, 300] class_weight: [None, ‘balanced’]
Extreme Gradient Boosting (XGB)	n_estimators: [50, 100, 200, 500] max_depth: [3, 6, 10] learning_rate: [0.01, 0.05, 0.1] subsample: [0.6, 0.8, 1.0] colsample_bytree: [0.6, 0.8, 1.0]
Gradient Boosting Machine (GBM)	n_estimators: [50, 100, 200] learning_rate: [0.01, 0.05, 0.1] max_depth: [3, 5, 7] min_samples_split: [2, 5, 10] subsample: [0.8, 0.9, 1.0]

## 3 Results

### 3.1 Clinical characteristics of patients

We enrolled 59 patients with acute myeloid leukemia (AML, non-M3), 34 patients who achieved complete bone marrow remission after treatment (AML-CR), and 101 individuals with normal bone marrow flow cytometry results (Norm). The dataset is randomly divided into a training set (n=135) and a test set (n=59) in a 7:3 ratio. The collected variables encompassed demographic characteristics (age and sex), routine blood parameters (white blood cell count, hemoglobin level, and platelet count), the proportion of bone marrow blasts/immunized cells, and 53 commonly used markers from flow cytometry data. The baseline characteristics of the participants are summarized in **Table 3**. The mean age of patients in the training set is 66.59 years (66.59 ± 14.33), while that in the test set is 66.66 years (66.66 ± 13.81), with no statistically significant difference between the two groups (P > 0.05). Chi-square analysis revealed no significant differences (P > 0.05) in any of the examined variables between the training and test sets, indicating similar distribution patterns across all factors. These results demonstrate that both the training and test sets are well-balanced and appropriate for subsequent predictive analysis.

**Table 3:** Clinical characteristics of the training set and test set.

Variables	Train set	Test set	Value of *t*	Value of *P*
Age	66.59 ± 14.33	66.66 ± 13.81	-0.03	0.97
Sex			-0.08	0.94
Male	77(57.04%)	34(57.63%)		
Female	58(42.96%)	25(42.37%)		
WBC (10^9/L)	17.25 ± 50.89	20.12 ± 61.81	-0.34	0.74
Hb(g/L)	87.70 ± 39.18	87.30 ± 37.42	0.07	0.95
PLT (10^9/L)	141.12 ± 163.65	127.41 ± 158.05	0.54	0.59
BMBC (%)	0.14 ± 0.24	0.15 ± 0.27	-0.31	0.76

WBC, white blood cell count; Hb, hemoglobin level; PLT, platelet count; BMBC, bone marrow blast cell count.

### 3.2 Model performance

**Figure 3:**
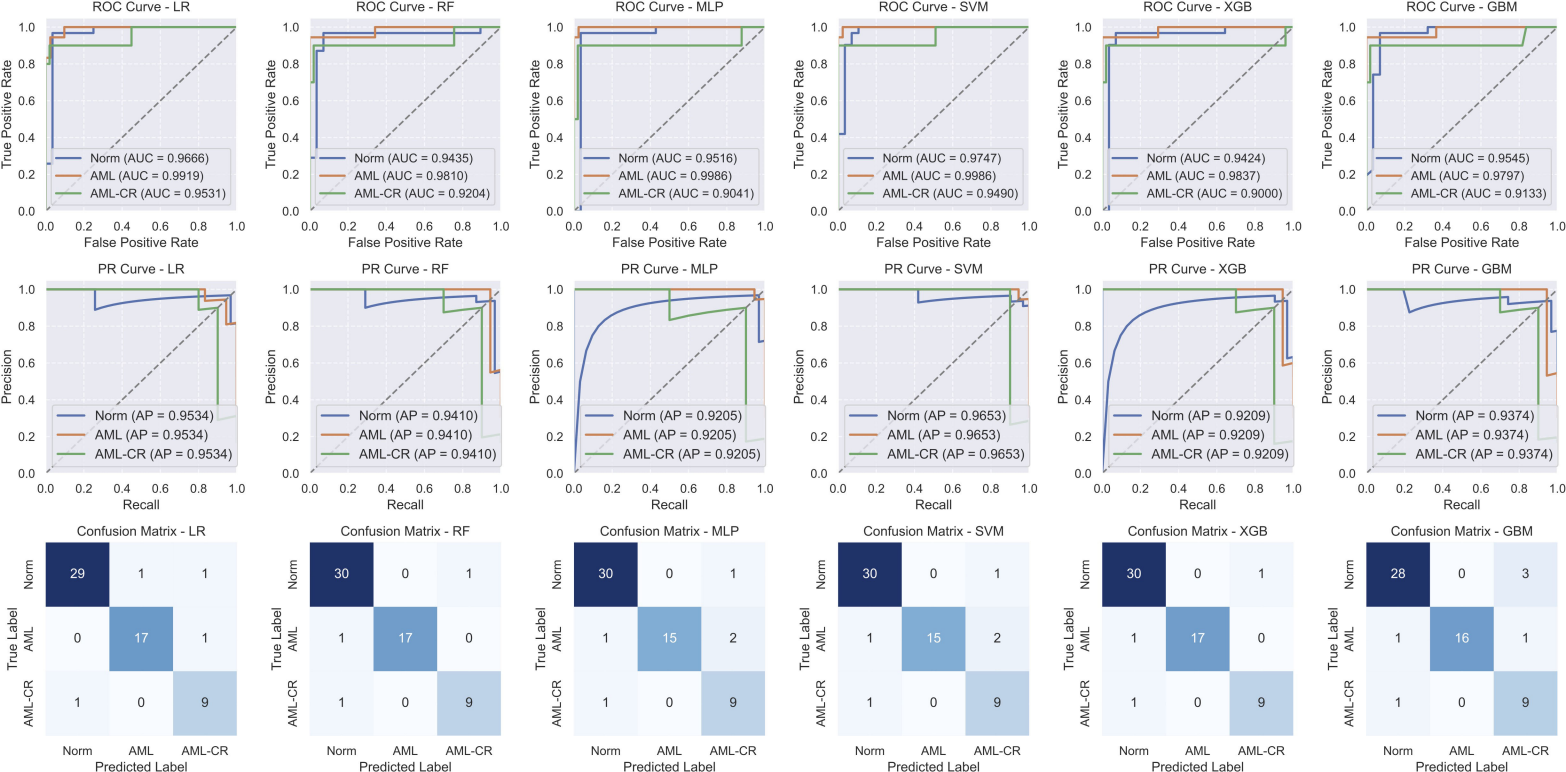
The performance analysis of different models. The first row shows the receiver operating characteristic (ROC) curve, the second row shows the precision-recall (PR) curve, and the third row shows the confusion matrix. LR, linear regression; RF, random forest; MLP, multi-layer perceptron; SVM, support vector machine; XGB, extreme gradient boosting; GBM, gradient boosting machine.

**Table 4** and **Figure 3** present the performance of different models in the classification task. Overall, both RF and XGB achieved superior results across all metrics, with F1-score, precision, and recall reaching 0.9413, 0.9458, and 0.9374, respectively, and an accuracy of 0.9492. These results indicate strong generalization capabilities for these two models in the classification task. LR exhibited a high AUC value (0.9705); however, its F1-score (0.9175) and precision (0.9098) are slightly lower than those of RF and XGB, suggesting some degree of misclassification. Both MLP and SVM demonstrated identical classification performance, with an accuracy of 0.9153 and an F1 score of 0.8932. Notably, SVM achieved the highest AUC value (0.9741), although its other metrics are lower than those of RF and XGB. GBM performed slightly worse than the other models, with the lowest accuracy (0.8983) and F1 score (0.8806). Although its AUC value reached 0.9491, its precision (0.8752) and recall (0.8974) exhibited a certain gap, possibly due to the model’s limited ability to distinguish between specific classes. Overall, RF and XGB demonstrated robust performance across all metrics, making them the most suitable candidates for this task. Meanwhile, SVM, with the highest AUC value, may offer advantages in certain application scenarios. Additionally, confusion matrix analysis revealed that the misclassified samples are evenly distributed across different categories, indicating that these models maintained a balanced classification error across classes.

**Table 4:** The performance of different models.

Model	Accuracy (%)	F1(%)	Precision (%)	Recall (%)	AUC (%)
LR	93.22	91.75	90.98	92.66	97.05
RF	94.92	94.13	94.58	93.74	94.83
MLP	91.53	89.32	89.58	90.04	95.14
SVM	91.53	89.32	89.58	90.04	97.41
XGB	94.92	94.13	94.58	93.74	94.2
GBM	89.83	88.06	87.52	89.74	94.91

LR, linear regression; RF, random forest; MLP, multi-layer perceptron; SVM, support vector machine; XGB, extreme gradient boosting; GBM, gradient boosting machine.

### 3.3 Feature importance analysis

**Figure 4:**
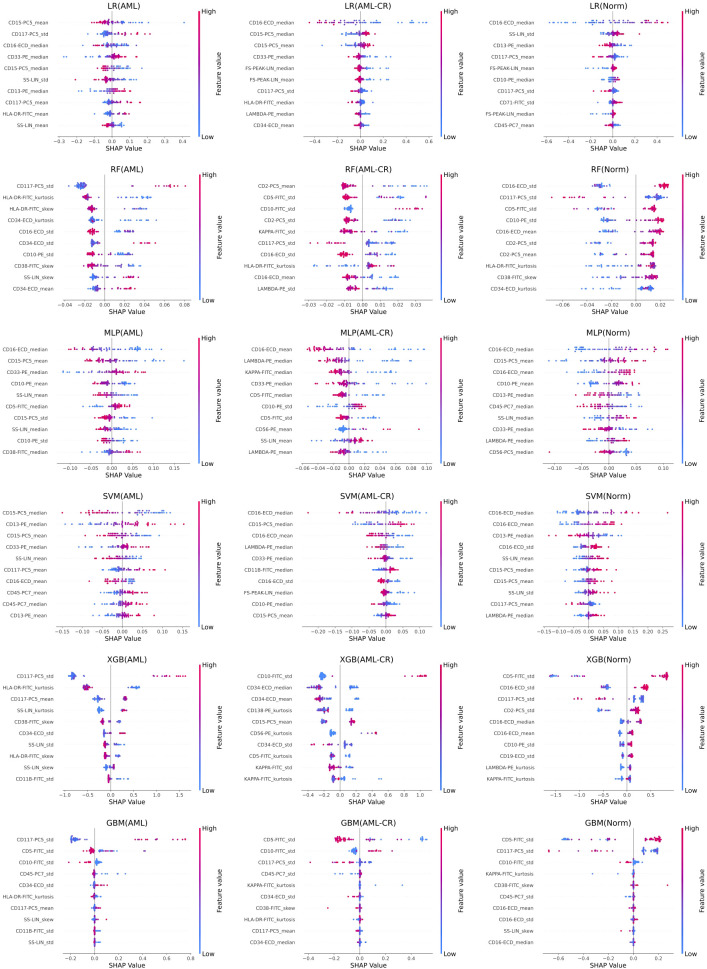
The feature importance analysis of different models based on SHAP. Each row represents the SHAP feature importance analysis results of a method on acute myeloid leukemia (AML), acute myeloid leukemia complete remission (AML-CR), and normal (Norm) classes, respectively. LR, linear regression; RF, random forest; MLP, multi-layer perceptron; SVM, support vector machine; XGB, extreme gradient boosting; GBM, gradient boosting machine.

**Figure 4** displays the top 10 most important features in the decision-making process for each model. Notably, CD117 and CD34 are identified as the most influential markers for distinguishing AML in the GBM, RF, and XGB models. These findings resonate with clinical practice, where both CD117 and CD34 are extensively utilized for AML differentiation and classification. This congruence between model predictions and clinical practices underscores the potential of data-driven approaches in medical diagnostics and highlights the pivotal roles of CD117 and CD34 in AML diagnosis. HLA-DR significantly affects the classification of AML and AML-CR in the GBM, RF, and LR models, while CD45 plays a crucial role in the GBM and SVM models, suggesting its potential contribution to the regulation of immunophenotypic heterogeneity in AML. Lower expression of HLA-DR is commonly observed in immature leukemia cells, particularly in M0/M1 subtypes, indicating a differentiation block that may facilitate immune evasion or serve as a marker for specific stages of differentiation. Furthermore, CD45 expression varies throughout the stages of myeloid differentiation, with its heterogeneity, in conjunction with differential expression of HLA-DR, contributing to the formation of different immune subtypes, thus indicating a differentiation blockade and the coexistence of multiple stages of differentiation. Additionally, the markers in the Norm group exhibit lower specificity, raising concerns about the risk of overfitting in some models due to the smaller phenotypic variability observed in normal samples. These insights suggest that while the models generally perform well, caution is warranted regarding the risk of overfitting, especially when distinguishing between normal and diseased states.

## 4 Discussion

Acute myeloid leukemia (AML) is a highly aggressive and heterogeneous hematopoietic malignancy, representing 15-20% of all leukemia diagnoses worldwide. The global annual incidence is estimated at 1.0–1.5 per 100,000 population, corresponding to approximately 40,000–50,000 new cases each year, with around 80% occurring in adults (aged ≥ 18 years) (25). Despite significant advances in therapeutic modalities, including chemotherapy, allogeneic stem cell transplantation, and novel targeted/immunotherapeutic agents, the overall prognosis of AML remains unsatisfactory. Patients with high-risk features (e.g., advanced age [≥60 years], TP53 mutations, or complex karyotypes) exhibit particularly dismal outcomes, with 5-year overall survival rates as low as 5-15% (4). The therapeutic landscape remains particularly constrained for patients with relapsed/refractory (R/R) AML. Disease progression is frequently complicated by life-threatening cytopenias (including transfusion-dependent anemia), opportunistic infections, and progressive multiorgan dysfunction - clinical manifestations directly attributable to the intrinsically aggressive biology of leukemic cells. This underscores the critical unmet need for advanced diagnostic modalities to guide precision therapeutic strategies. Flow cytometry-based immunophenotyping analysis offers critical support for the early detection of disease progression and the development of personalized treatment strategies by dynamically monitoring cell characteristics.

Conventional flow cytometry methods for manual detection typically rely on gating and clustering techniques to categorize cells into multiple subpopulations, performing multi-parameter analysis of surface and intracellular markers. These methods are widely used for the diagnosis and classification of hematologic malignancies (26). Conventional flow cytometry data analysis remains labor-intensive and subject to inter-operator variability due to its reliance on manual gating expertise. This inherent limitation has spurred the rapid adoption of machine learning algorithms in hematological diagnostics, enabling automated analysis of cellular morphology, immunophenotypic patterns, and histopathological features with enhanced reproducibility (27–29). Among them, Beni et al. (30) introduces a multi-cell classification benchmark dataset; Hu et al. (31) uses deep convolutional neural networks for cytomegalovirus classification based on flow cytometry data; Li et al. (32) transforms SW-480 epithelial cancer cell flow cytometry data into images and uses convolutional neural networks for classification. Although these methods have positively contributed to the improvement of flow cytometry diagnostics, their practical implementation continues to encounter substantial challenges: (1) the inherently high-dimensional nature of patient-level FCM data; (2) substantial inter-sample variability introduced by both biological heterogeneity (e.g., treatment response status) and technical factors (e.g., instrument configuration); and (3) the critical knowledge gap regarding immunophenotypic patterns during remission phases. Addressing these challenges, we developed a novel cell-level data integration framework using real-world clinical FCM datasets. Notably, our study represents the first systematic incorporation of remission phase AML samples into classification models, thereby establishing a much-needed benchmark for treatment response monitoring.

This study pioneers the inclusion of AML patients in complete remission (AML-CR) within a classification system and has successfully developed an artificial intelligence model that accurately differentiates among healthy individuals, AML patients, and AML-CR. The model demonstrated > 90% accuracy in all baseline tests, confirming its validity. This high accuracy further indicates that AML-CR exhibits significantly distinct characteristics compared to the other two groups, offering new perspectives for clinical diagnosis. Notably, our machine learning model shows substantial speed advantages over both manual analysis and deep learning methods. In bone marrow flow cytometry testing, the entire process from sample processing and staining to data acquisition typically requires several to over ten minutes (33). Data analysis requires professionals to manually gate and analyze antigen expression patterns while integrating clinical background for interpretation. Flow cytometry specialists at Shanghai KingMed Diagnostics report that the analytical duration varies significantly (15 minutes to several hours) depending on sample complexity, clinical requirements, and operator experience. Our diagnostic approach completes single-patient data analysis in< 1 second, demonstrating two key advantages: (1) a 100-1000× improvement in processing speed compared to conventional methods, and (2) a substantial reduction in technologist workload. Clinical implementation of this method enables real-time assessment of disease status and treatment response, supporting timely therapeutic decision-making. By applying the proposed method to actual flow cytometry data analysis, physicians can evaluate patients’ disease status and treatment response more rapidly and accurately, thereby developing more personalized treatment plans. AI-assisted flow cytometry analysis is expected to play an important role in primary medical institutions lacking flow cytometry diagnostic specialists, helping more patients benefit.

Multimodal SHAP analysis demonstrates that the key markers of the AML group (CD117, CD34, HLA-DR) exhibit strong concordance with established clinical diagnostic criteria, thereby validating their pivotal role as core immunophenotypic markers for leukemia cell identification and classification. We identified significant differences in the expression patterns of specific cell surface markers among *de novo* patients with AML, healthy individuals, and post-treatment AML patients who achieved complete remission. Notably, CD34 and CD117 expression levels are significantly higher in AML patients compared to both healthy individuals and remission-phase patients, whereas CD45 expression is comparatively reduced. These findings suggest a potential mechanistic link between aberrant marker expression and AML pathogenesis, therapeutic efficacy, and relapse, advancing our understanding of the disease’s biology. Furthermore, flow cytometric profiling of AML patients in complete remission facilitates the identification of therapy-responsive immunological markers and provides early warning signs for potential disease recurrence. Notably, the markers identified in the normal group demonstrated relatively low specificity, potentially reflecting physiological variations in immune homeostasis. To improve model robustness, adversarial training or sample size expansion approaches should be considered to enhance interference resistance.

Notwithstanding the meaningful contributions of this work, certain limitations merit consideration. Chief among these is the restricted generalizability inherent to single-center studies with limited sample sizes. Second, the intrinsic heterogeneity and technical variability in flow cytometry data may introduce measurement noise and analytical interference, which could adversely affect the model’s predictive accuracy. Additionally, the biological implications of specific cell surface markers warrant further investigation. Future studies should employ expanded cohorts incorporating diverse AML subtypes and treatment phases to optimize and validate the machine learning model’s performance. Furthermore, integrating genomic sequencing data and other multi-omics information to develop a multi-omics fusion model, alongside multimodal imaging data and clinical information, could provide a more comprehensive AML diagnostic and prognostic assessment tool, thereby advancing the precision and scientific rigor of clinical decision-making. The current study is limited to non-M3 AML patients. Future research should extend the model’s applicability to additional AML subtypes, including acute promyelocytic leukemia (M3) and other rare variants, to enhance its clinical utility. Looking forward, translational application of this model to the broader spectrum of hematologic neoplasms, such as acute lymphoblastic leukemia, may establish new frameworks for AI-powered.

## Data Availability

The original contributions presented in the study are included in the article/supplementary material. Further inquiries can be directed to the corresponding authors.
